# Early variations of laboratory parameters predicting shunt-dependent hydrocephalus after subarachnoid hemorrhage

**DOI:** 10.1371/journal.pone.0189499

**Published:** 2017-12-12

**Authors:** Min Kyun Na, Yu Deok Won, Choong Hyun Kim, Jae Min Kim, Jin Hwan Cheong, Je il Ryu, Myung-Hoon Han

**Affiliations:** Department of Neurosurgery, Hanyang University Guri Hospital, Gyeongchun-ro, Guri, Gyonggi-do, Korea; Universidad Miguel Hernandez de Elche, SPAIN

## Abstract

**Background and purpose:**

Hydrocephalus is a frequent complication following subarachnoid hemorrhage. Few studies investigated the association between laboratory parameters and shunt-dependent hydrocephalus. This study aimed to investigate the variations of laboratory parameters after subarachnoid hemorrhage. We also attempted to identify predictive laboratory parameters for shunt-dependent hydrocephalus.

**Methods:**

Multiple imputation was performed to fill the missing laboratory data using Bayesian methods in SPSS. We used univariate and multivariate Cox regression analyses to calculate hazard ratios for shunt-dependent hydrocephalus based on clinical and laboratory factors. The area under the receiver operating characteristic curve was used to determine the laboratory risk values predicting shunt-dependent hydrocephalus.

**Results:**

We included 181 participants with a mean age of 54.4 years. Higher sodium (hazard ratio, 1.53; 95% confidence interval, 1.13–2.07; p = 0.005), lower potassium, and higher glucose levels were associated with higher shunt-dependent hydrocephalus. The receiver operating characteristic curve analysis showed that the areas under the curve of sodium, potassium, and glucose were 0.649 (cutoff value, 142.75 mEq/L), 0.609 (cutoff value, 3.04 mmol/L), and 0.664 (cutoff value, 140.51 mg/dL), respectively.

**Conclusions:**

Despite the exploratory nature of this study, we found that higher sodium, lower potassium, and higher glucose levels were predictive values for shunt-dependent hydrocephalus from postoperative day (POD) 1 to POD 12–16 after subarachnoid hemorrhage. Strict correction of electrolyte imbalance seems necessary to reduce shunt-dependent hydrocephalus. Further large studies are warranted to confirm our findings.

## Introduction

Hydrocephalus is one of the frequent complications after recovering from subarachnoid hemorrhage (SAH). The incidence of hydrocephalus after SAH is approximately 6 to 67%.[1,2] Although they are not fully established, some possible pathophysiological mechanisms include deterioration of cerebrospinal fluid (CSF) dynamics, obstructive mechanisms by blood products, and impaired CSF absorption at the damaged arachnoid granulation level.[3,4]

Among hydrocephalus patients, about 10 to 20% patients require permanent CSF diversion.[5] Previous studies reported the risk factors associated with hydrocephalus after SAH including old age, female sex, high Hunt-Hess grade on admission, high modified Fisher grade, presence of intraventricular hemorrhage (IVH), external ventricular drainage (EVD), and meningitis.[5–9] However, few studies investigated the association between laboratory parameters and shunt-dependent hydrocephalus.

The purpose of this study was to investigate the variations of laboratory parameters within 28 days after clipping due to aneurysmal SAH. In addition, we attempted to identify predictive laboratory parameters for shunt-dependent hydrocephalus with estimation of cut-off values for each laboratory value.

## Methods

### Study patients

We retrospectively searched for patients (>18 years old) with SAH diagnosis codes of I60-I609, based on the International Classification of Diseases, tenth edition (ICD-10). The patients were admitted to our hospital from October 1, 2008, to December 31, 2016. Among these patients, we extracted those with procedure codes for clipping (HN 46400, simple; HN46420, complex).

Due to preference to clipping in SAH patients in our hospital, we excluded few coiling cases. We think this may reduce possible effect of treatment heterogeneity between clipping and coiling on variations of the laboratory parameters. A total of 235 patients were initially identified. We excluded 54 patients from this study who met any of the following criteria: (1) expired within 28 days after aneurysmal clipping, (2) postoperative hemorrhagic complications and, (3) coding errors. Finally, the remaining 181 patients who underwent aneurysmal clipping for spontaneous SAH due to a ruptured aneurysm were included in this study. All aneurysmal clippings were performed within 24 hours of SAH diagnosis in the emergency room. V-P shunts were performed in the patients who had hydrocephalus-related symptoms, such as altered consciousness, gait disturbance, and urinary incontinence with the presence of ventricular enlargement on CT scans. We also performed V-P shunts in patients not tolerating CSF drainage weaning who maintained EVD chronically.

This study was approved by the Institutional Review Board of Hanyang University Medical Center. Due to the retrospective nature of this study, the ethics committee did not require subsequent informed written consent from patients. However, we de-identified and anonymized patient records prior to analysis. We provide all relevant study data in S1 Data.

### Operation and management

All patients were treated using our institutional SAH treatment algorithm, which includes intracranial pressure control with mannitol and glycerol, anticonvulsant agent, triple-H (hypertension/hypervolemia/hemodynamic) therapy, and administration of nimodipine. All operations were performed by two neurosurgeons. According to the laboratory values during follow-up after surgery, we corrected blood cell count, electrolyte, glucose and albumin imbalance.[10] The normal values of laboratory parameters are as follows: (1) hemoglobin, 10.0–16.0 g/dL; (2) platelet, 140.0–400.0 x 103/mm3; (3) sodium, 135–145 mEq/L; (4) potassium, 3.5–5.0 mEq/L; (5) glucose (fasting), 70–126 mg/dL; (6) albumin, 3.5–5.2 g/dL. All parameter imbalances were corrected when the estimated values exceeded the upper and lower limit. Packed RBC transfusion was administrated to maintain a hemoglobin concentration above 8–10 g/dL. All patients routinely received 0.9% saline (1000 ml) and a complete supply of total parenteral nutrition (TPN) each day after admission with no potassium added during their intensive care unit (ICU) stay. If the serum sodium level was under 135 mEq/L, 300 mL of 3% saline was administered and the normal saline dose was reduced by the same volume. Moreover, if the serum sodium level was over 145 mEq/L, intravenous normal saline fluid was replaced by 0.45% saline and we increased oral water intake. If the serum potassium level was under 3.5 mEq/L, 20 mM potassium was added to the intravenous saline fluid. If the serum potassium level was under 3.0 mEq/L, 40 mM potassium was added, and oral potassium was administered. We measured serum glucose four times a day to avoid hypoglycemia (serum glucose <80 mg/dL) and hyperglycemia (serum glucose > 200 mg/dL and > 126 mg/dL in fasting state), and insulin was administrated to maintain serum glucose below 200 mg/dL and 126 mg/dL (fasting).

Surgical techniques and patient management were identical for both surgeons (a junior neurosurgeon was trained and supervised in surgical techniques and management for SAH by a senior neurosurgeon). No patient was treated with lumbar CSF drainage insertion. Our department policy avoids this procedure to prevent brain herniation in patients with intracranial hypertension.

### Clinical and radiographic variables

Clinical and demographic data were retrospectively reviewed using electronic medical records of all patients, including patient admission and discharge status, date of V-P shunt insertion, and length of follow-up. Hunt-Hess grade was used to determine the neurological status of patients and severity of SAH.[11] The CT scans at admission were obtained with a CT scanner (Siemens Flash 128, München, Germany) and were evaluated for the thickness of SAH, presence of intracerebral hemorrhage (ICH), presence of IVH (no, focal, pan ventricle), location of aneurysm, and modified Fisher grade.[12] Pan ventricular hemorrhage was defined by the presence of hematoma in both the lateral, and the third and fourth ventricles simultaneously. All CT scans were reviewed by two experienced neurosurgeons blinded to the clinical data of patients.

### Laboratory test results and missing values

Laboratory parameters were retrieved from our hospital database. We obtained laboratory results immediately upon admission (preoperative) and subsequently during hospital stay (immediate postoperative and postoperative day (POD) 1, 2, 3, 4, 6–8, 12–16, 21–28). Blood samples were obtained in fasting states in the morning since POD 1. We investigated the levels of laboratory parameters, including white blood cell, hemoglobin, platelet, sodium, potassium, glucose, blood urine nitrogen (BUN), creatinine, osmolarity, and albumin. We performed multiple imputation to fill the missing laboratory data using Bayesian methods in SPSS.[13] The minimum and maximum options were set to minimum and maximum values for each laboratory parameter to avoid imputed values that were negative.[14] The information of each laboratory missing value is presented in S1 and S2 Figs.

### Statistical methods

Patients were divided into two groups based on whether V-P shunt was performed after clipping for aneurysmal ruptured SAH occurrence. Continuous variables are presented as the mean ± standard deviation, and discrete variables as count with percentage. The Chi-square test and Student’s t-test were performed to identify clinical differences between the no shunt treated group and shunt-dependent hydrocephalus group.

The receiver operating characteristic (ROC) curve was used to determine the laboratory parameters from POD 1 to POD 12–16 for predicting shunt-dependent hydrocephalus in patients with SAH. The laboratory parameters with the highest sensitivity and specificity were defined as the optimal cutoff value. We performed the Kaplan-Meier analysis to evaluate the predictive factors for shunt-dependent hydrocephalus, with censoring of patients who were lost during follow-up. The univariate Cox regression analysis was used to calculate hazard ratios (HRs) with 95% confidence intervals (CIs) for the shunt-dependent hydrocephalus, based on clinical and laboratory values. Subsequently, sex, age, and covariates that were significant at p < 0.05 in the univariate analysis were included in a multivariate analysis to identify independent factors associated with shunt-dependent hydrocephalus after clipping for SAH.

## Results

### Patient characteristics

A total of 181 consecutive patients (>18 years old) with spontaneous SAH due to a ruptured aneurysm and who underwent clipping were enrolled over a 9-year period at our hospital. The mean patient age was 54.4 years, and 106 patients (58.6%) were female. Shunt-dependent hydrocephalus occurred in 43 patients (23.8%). The median time interval between SAH and shunting was 51 days (IQR: 32 to 72) and the earliest shunt operation was performed at POD 10 in the study (data not shown). Significant differences were found in age, intensive care unit (ICU) stay, Hunt-Hess grade, IVH, and EVD prior to V-P shunt between shunt-dependent hydrocephalus and no shunt treated groups. Further descriptive data are shown in Table 1.

**Table 1:** Characteristics of patients with spontaneous subarachnoid hemorrhage who underwent aneurysmal clipping classified by ventriculo-peritoneal shunt operation.

Characteristics	No shunt treated group (n = 138)	Shunt-dependent hydrocephalus (n = 43)	Total (n = 181)	P
Sex				0.640
Female, n (%)	79 (57.2)	27 (62.8)	106 (58.6)	
Age, mean ± SD, y	52.9 ± 11.4	59.0 ± 11.3	54.4 ± 11.6	0.003
Age range, y	28–82	39–78	28–82	
Age group, n (%)				0.022
<65	117 (84.8)	29 (67.4)	146 (80.7)	
≥65	21 (15.2)	14 (32.6)	35 (19.3)	
Time interval between SAH and shunting, median (IQR), day		51 (32–72)		
ICU stay, median (IQR), day	6 (4–10)	19 (7–32)	7 (4–17)	< 0.001
Follow-up period, median (IQR), month	26.1 (8.5–61.2)	32.0 (12.0–55.3)	26.5 (9.4–56.9)	0.432
Hunt-Hess grade, n (%)				0.011
Grade 1	7 (5.1)	1 (2.3)	8 (4.4)	
Grade 2	75 (54.3)	12 (27.9)	87 (48.1)	
Grade 3	37 (26.8)	16 (37.2)	53 (29.3)	
Grade 4	17 (12.3)	13 (30.2)	30 (16.6)	
Grade 5	2 (1.4)	1 (2.3)	3 (1.7)	
Aneurysm location, n (%)				0.215
ACA	42 (30.4)	21 (48.8)	63 (34.8)	
ICA	9 (6.5)	3 (7.0)	12 (6.6)	
MCA	50 (36.2)	9 (20.9)	59 (32.6)	
PCOM	32 (23.2)	9 (20.9)	41 (22.7)	
VBA	5 (3.6)	1 (2.3)	6 (3.3)	
Modified Fisher grade, n (%)				0.283
1	33 (23.9)	7 (16.3)	40 (22.1)	
2	23 (16.7)	6 (14.0)	29 (16.0)	
3	31 (22.5)	7 (16.3)	38 (21.0)	
4	51 (37.0)	23 (53.5)	74 (40.9)	
IVH, n (%)				0.014
No	64 (46.4)	14 (32.6)	78 (43.1)	
Focal	49 (35.5)	12 (27.9)	61 (33.7)	
Pan ventricle	25 (18.1)	17 (39.5)	42 (23.2)	
ICH present, n (%)	39 (28.3)	9 (20.9)	48 (26.5)	0.451
EVD prior to V-P shunt, n (%)	5 (3.6)	7 (16.3)	12 (6.6)	0.004
Craniectomy, n (%)	17 (12.3)	10 (23.3)	27 (14.9)	0.130

SD, standard deviation; ICU, intensive care unit; ACA, anterior cerebral artery; ICA, internal carotid artery; MCA, middle cerebral artery; PCOM, posterior communicating artery; VBA, vertebrobasilar artery; IVH, intraventricular hemorrhage; ICH, intracerebral hemorrhage; EVD, extra-ventricular drainage; V-P, ventriculo-peritoneal

### Association between shunt-dependent hydrocephalus and laboratory values

**Fig 1:**
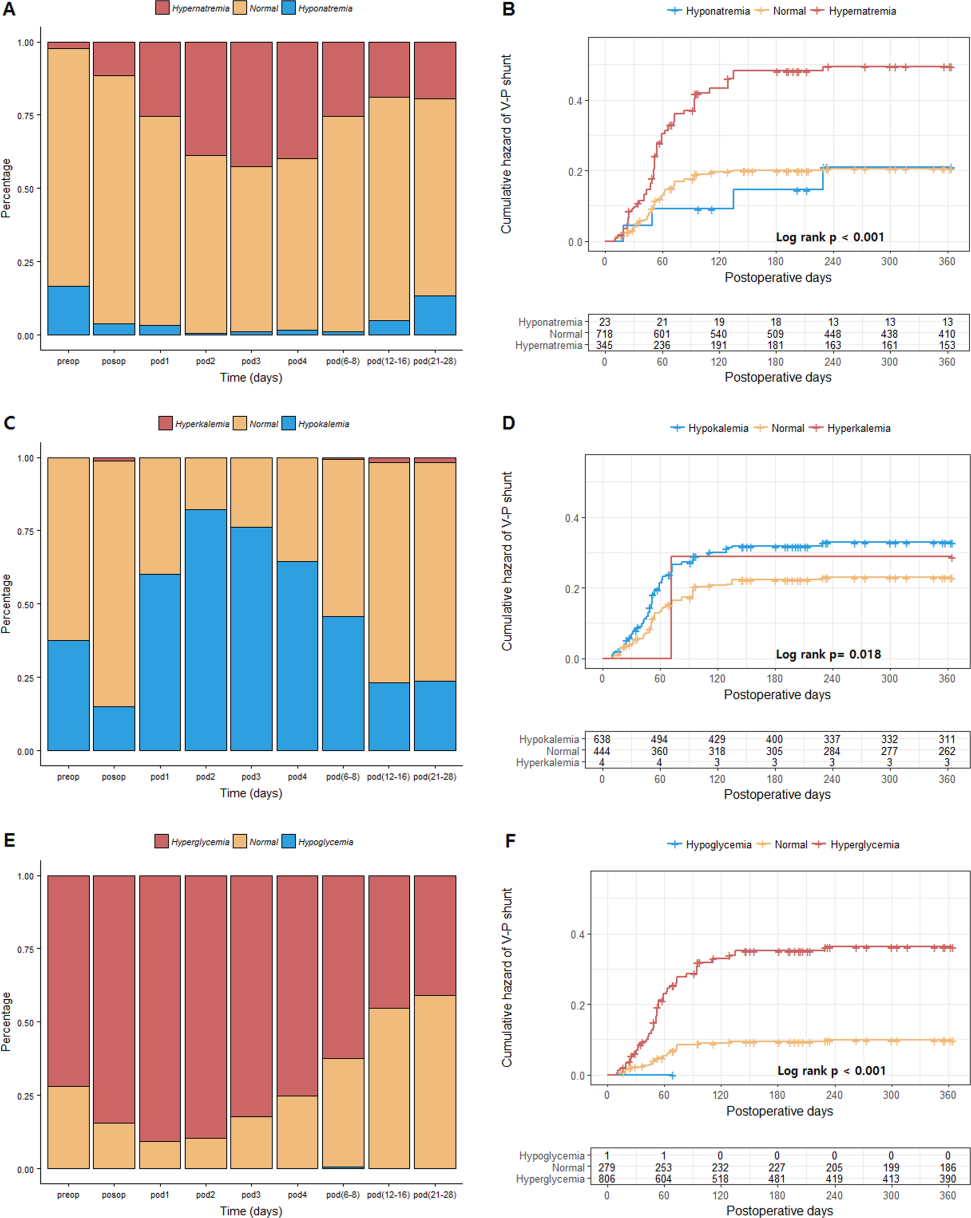
(A, C, E) Bar plot showing the variations of laboratory parameters after SAH occurrence. A, sodium (normal: 135–145 mEq/L); C, potassium (normal: 3.5–5.0 mmol/L); E, fasting glucose except for preoperative glucose (normal 70–126 mg/dL). (B, D, F) Kaplan-Meier curves showing the cumulative hazard ratio of V-P shunt after clipping for SAH according to laboratory parameters from POD 1 to POD 12–16. B, sodium; D, potassium; F, glucose.

The mean and standard deviation (SD) for laboratory parameters were estimated between the period from preoperative day to POD between 21 and 28 (Table 2). The mean serum sodium level at admission was 137.8 ± 4.2 mEq/L. Following surgery, the mean sodium levels increased gradually and showed the highest peak at POD 3 (144.8 ± 5.7 mEq/L). The mean glucose level at admission was 155.9 ± 47.0 mg/dL and showed peak at POD 1 (192.9 ± 63.9 mg/dL). Hyperglycemic conditions were maintained until POD 21–28 after SAH occurrence. The mean potassium level at admission was 3.5 ± 0.4 mmol/L, and the lowest level of potassium was observed at POD 2 and 3 (3.2 ± 0.4 mmol/L). A higher proportion of patients presented hypernatremia, hypokalemia, and hyperglycemia in the early period after SAH occurrence (Fig 1A, 1C and 1E).

**Table 2:** Variations of laboratory parameters as time increases in patients with subarachnoid hemorrhage who underwent aneurysmal clipping.

Valuables	Laboratory data, mean (SD)								
	Preoperation (n = 181)	Postoperation (n = 181)	POD 1 (n = 181)	POD 2 (n = 181)	POD 3 (n = 181)	POD 4 (n = 181)	POD 6–8 (n = 181)	POD 12–16 (n = 181)	POD 21–28 (n = 181)
White blood cell (10^3^/mm^3^)	11.4 (4.1)	13.9 (4.8)	15.6 (4.8)	16.6 (5.6)	14.0 (5.4)	12.3 (5.8)	12.5 (4.1)	11.1 (4.5)	8.3 (4.4)
Hemoglobin (g/dl)	13.5 (1.7)	11.6 (1.5)	11.5 (1.5)	11.1 (1.5)	11.1 (1.3)	11.2 (1.2)	11.3 (1.3)	11.1 (1.2)	11.3 (1.2)
Platelet (10^3^/mm^3^)	243.8 (70.1)	193.6 (69.3)	180.2 (69.0)	166.2 (59.1)	167.6 (58.6)	179.6 (70.0)	207.3 (77.7)	294.7 (111.6)	255.1 (116.7)
Sodium (mEq/L)	137.8 (4.2)	140.2 (3.9)	141.9 (4.5)	144.5 (5.1)	144.8 (5.7)	144.7 (6.2)	142.7 (5.5)	140.4 (4.9)	140.6 (4.7)
Potassium (mmol/L)	3.5 (0.4)	3.9 (0.5)	3.3 (0.5)	3.2 (0.4)	3.2 (0.4)	3.5 (0.5)	3.8 (0.5)	3.8 (0.5)	3.9 (0.5)
Glucose (mg/dL)	155.9 (47.0)	164.3 (40.0)	192.9 (63.9)	173.2 (48.1)	163.3 (44.6)	155.7 (49.6)	149.7 (45.1)	131.3 (46.7)	128.8 (42.7)
Blood urine nitrogen (mg/dl)	14.5 (4.1)	12.6 (4.5)	16.0 (6.8)	18.7 (8.2)	18.8 (8.8)	18.9 (9.4)	19.5 (10.0)	14.9 (7.9)	14.3 (7.8)
Creatinine (mg/dl)	0.7 (0.2)	0.7 (0.2)	0.7 (0.3)	0.7 (0.3)	0.6 (0.3)	0.6 (0.3)	0.6 (0.3)	0.6 (0.2)	0.6 (0.2)
Osmolarity (mol/kg)	298.4 (11.1)	302.7 (10.3)	308.9 (14.8)	310.7 (12.1)	309.7 (13.5)	309.5 (14.7)	305.8 (16.2)	298.1 (13.5)	298.8 (12.8)
Albumin (g/dL)	4.3 (0.4)	3.1 (0.6)	3.4 (0.5)	3.5 (0.4)	3.4 (0.4)	3.5 (0.4)	3.4 (0.4)	3.4 (0.5)	3.6 (0.5)

SD, standard deviation; POD, postoperative day

**Fig 2:**
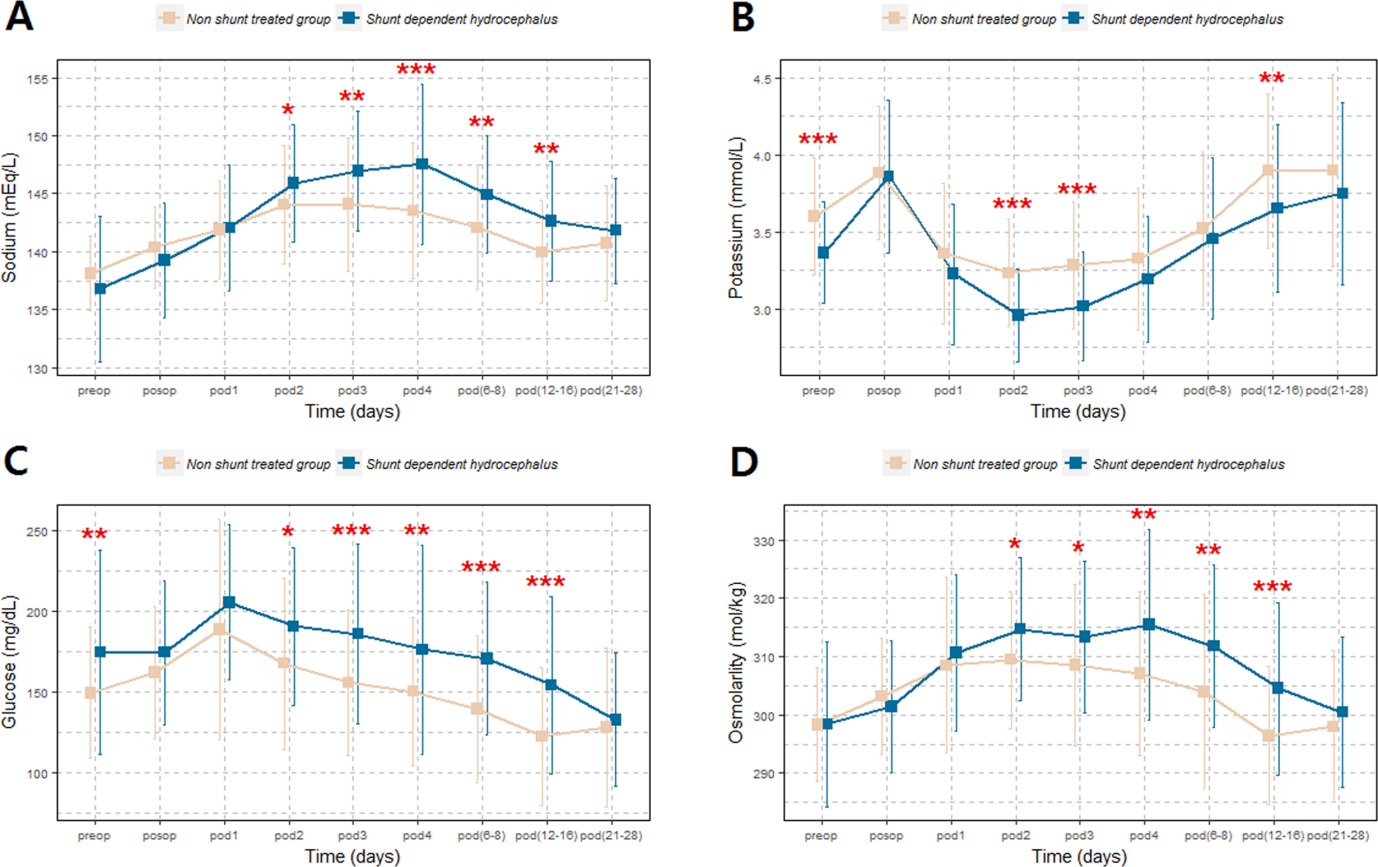
Variations of various laboratory parameters after subarachnoid hemorrhage occurrence based on shunt dependency: A, sodium; B, potassium; C, glucose; D, osmolarity. *p < 0.05, ** < 0.01, *** < 0.001.

We also present the laboratory data classified by the shunt operation in S1 Table. We compared the values of various laboratory parameters, from the preoperative day to POD 21–28, between shunt-dependent hydrocephalus and non-shunt treated groups. Significant differences were noted in the values of the laboratory parameters including sodium, potassium, glucose, and osmolarity, particularly between the period from POD 2 to POD 12–16 (Fig 2).

Shunt-dependent hydrocephalus patients showed significantly higher level of sodium from POD 2 to POD 12–16 compared with the non-shunt treated patients. The patients with low serum potassium level at admission and POD 2 and 3 showed higher rate of shunt-dependent hydrocephalus (Fig 1). In addition, the higher glucose levels at admission and from POD 2 to POD 12–16 were associated with shunt-dependent hydrocephalus. We also present variations of other laboratory values based on shunt dependency (S3 Fig). In addition, modified Fisher grades 2 and 4 and pan IVH showed a tendency of hypernatremia in S4 Fig.

**Fig 3:**
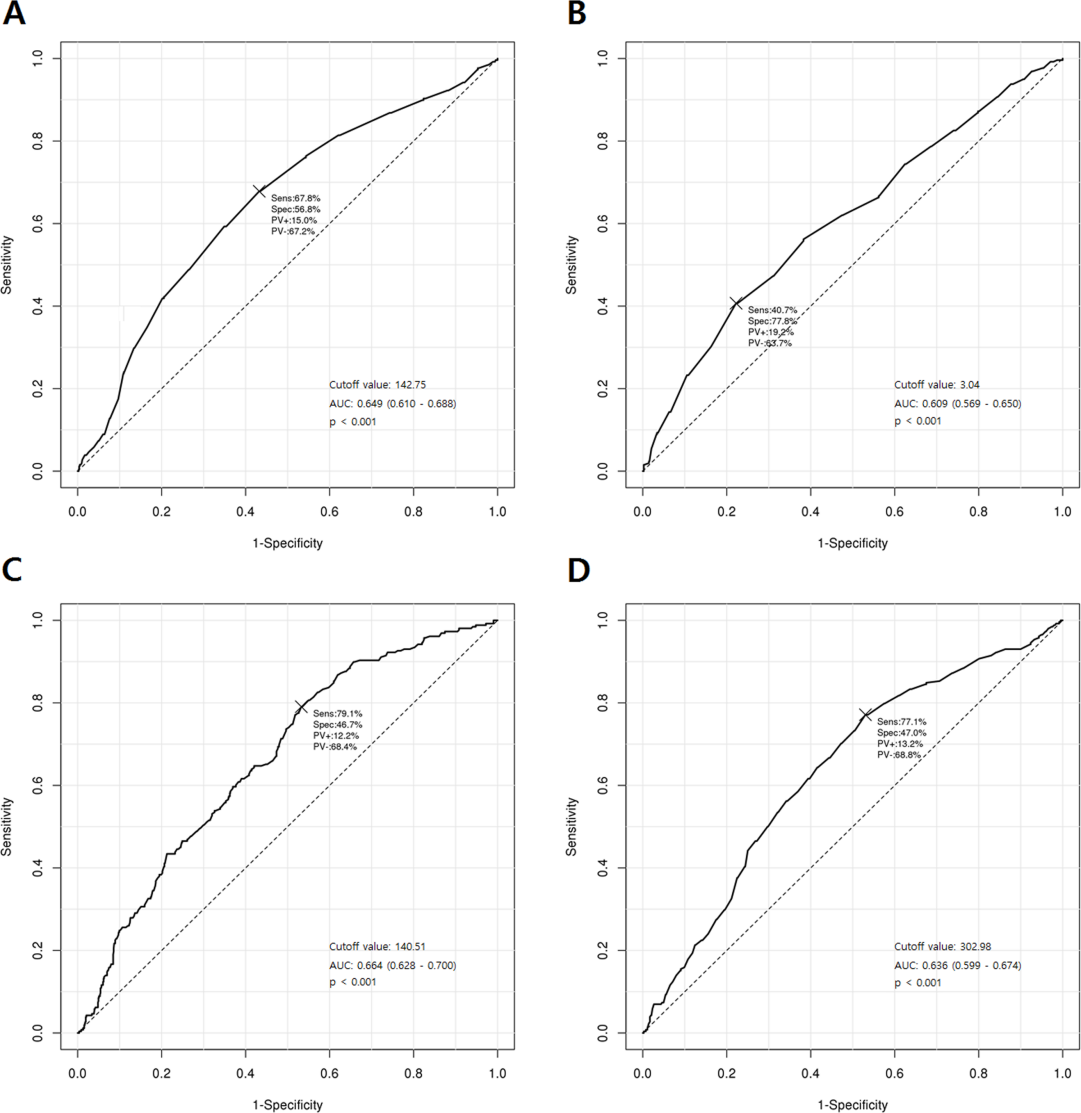
Receiver operating characteristic curve for shunt-dependent hydrocephalus after subarachnoid hemorrhage occurrence from POD 1 to POD 12–16 based on the following factors: A, sodium; B, potassium; C, glucose; D, osmolarity. POD = postoperative day.

The accuracy of laboratory factors for predicting shunt-dependent hydrocephalus was assessed by the ROC curve. On the ROC curve analysis, the areas under the curve of sodium, potassium, glucose, and osmolarity were 0.649 (95% CI, 0.610–0.688; p < 0.001; cutoff value, 142.75 mEq/L), 0.609 (95% CI, 0.569–0.650; p < 0.001; cutoff value, 3.04 mmol/L), 0.664 (95% CI, 0.628–0.700; p < 0.001; cutoff value, 140.51 mg/dL), and 0.636 (95% CI, 0.599–0.674; p < 0.001; cutoff value, 302.98 mol/kg), respectively, from POD 1 to POD 12–16 (Fig 3).

We present the ROC curves for shunt-dependent hydrocephalus based on other laboratory factors in S5 Fig.

**Fig 4:**
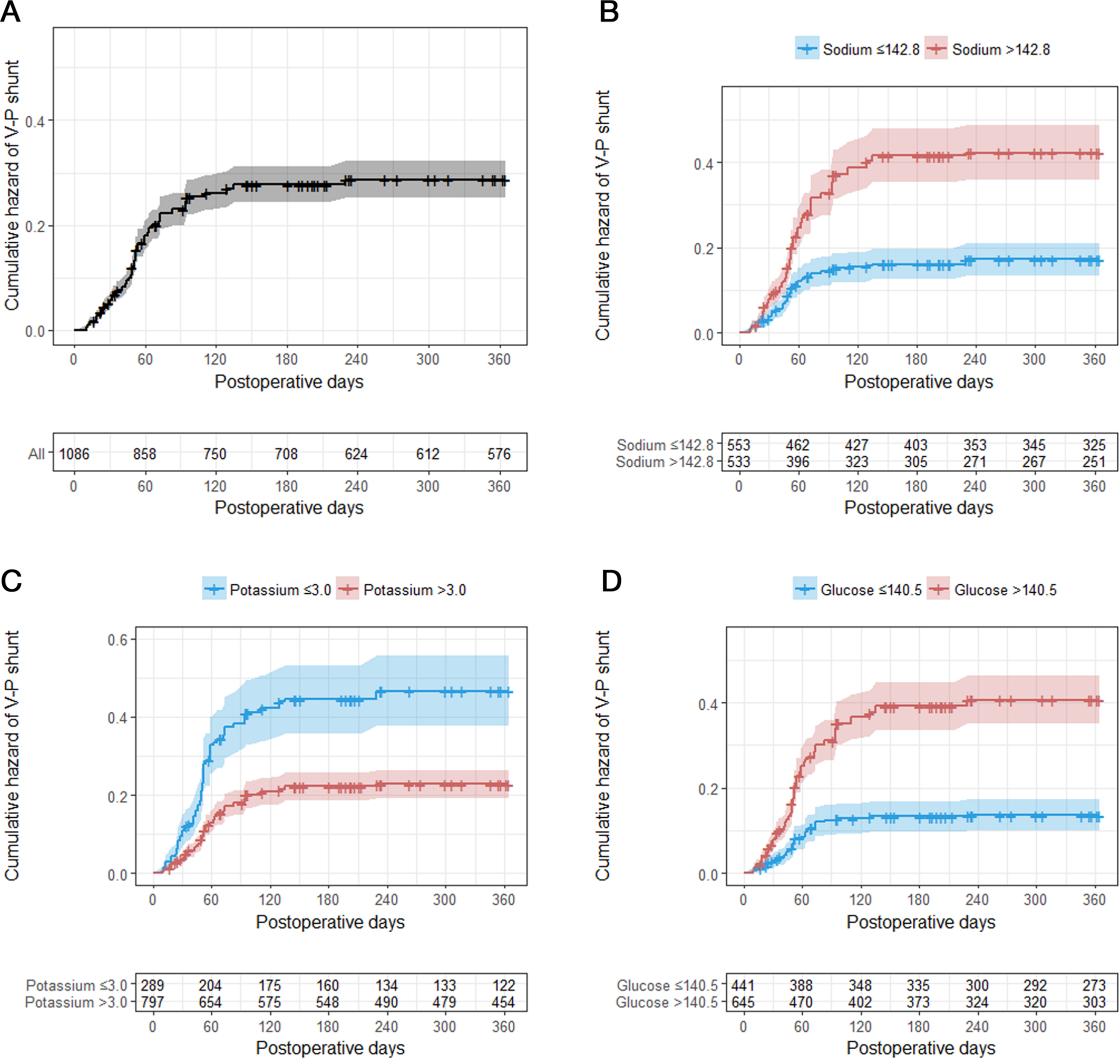
Kaplan-Meier curves showing the cumulative hazard ratio of V-P shunt after clipping for SAH according to several laboratory parameters from POD 1 to POD 12–16. A, Total; B, sodium; C, potassium; D, glucose. V-P = ventriculo-peritoneal; SAH = subarachnoid hemorrhage; POD = postoperative day.

We observed a higher shunt-dependent hydrocephalus in the higher sodium (>142.8 mEq/L), lower potassium (≦3.0 mmol/L), and higher glucose (>140.5 mg/dL) groups, which were classified by the cut-off values of the ROC curve, in the Kaplan-Meier analysis (Fig 4).

Hypernatremia from POD 1 to POD 12–16 showed higher shunt-dependent hydrocephalus (Fig 1B, 1D and 1F). Hypokalemia and hyperglycemia also showed higher shunt-dependent hydrocephalus.

### Clinical and laboratory factors related to shunt-dependent hydrocephalus after SAH

**Fig 5:**
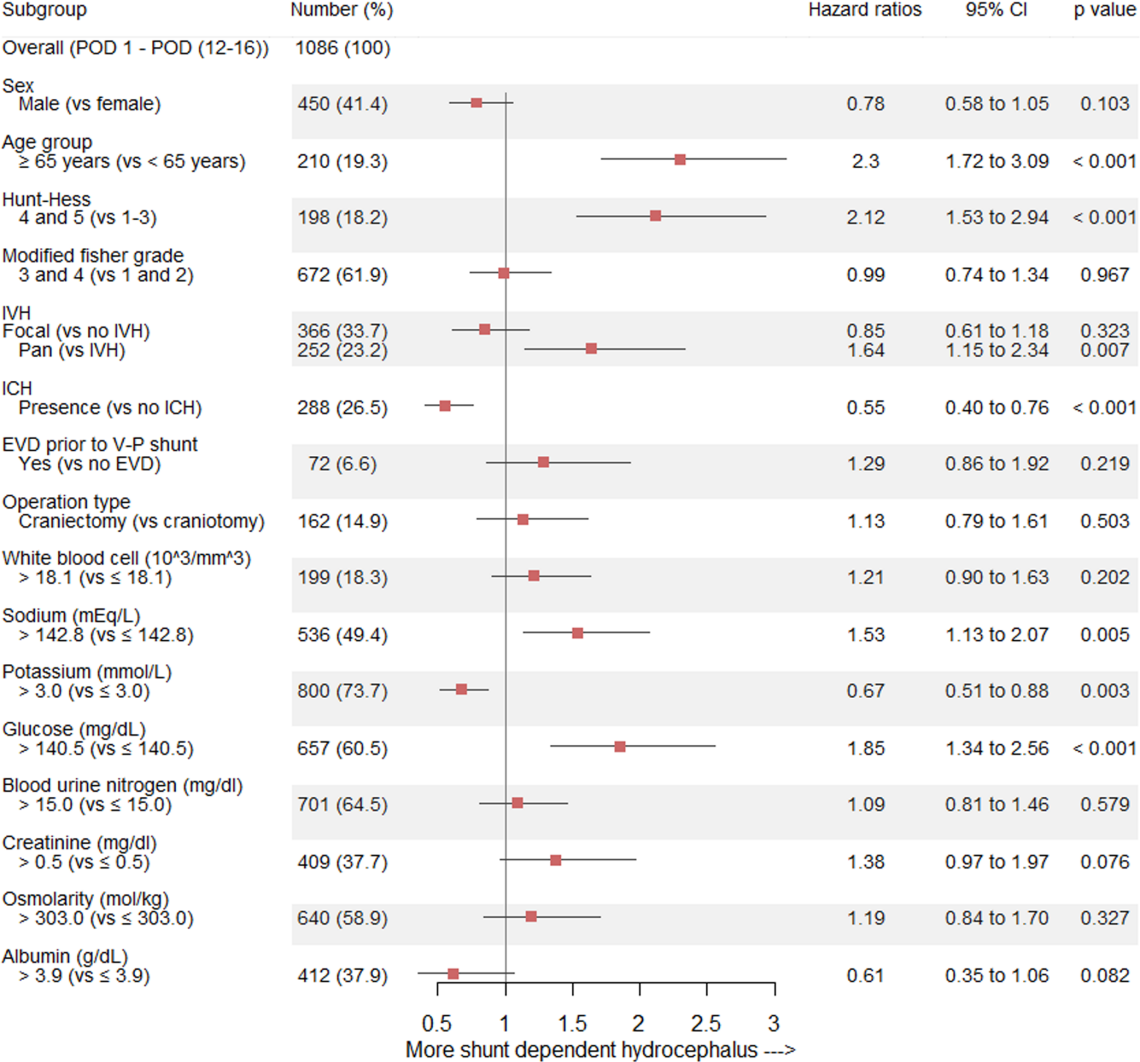
Forest plot of estimates from the multivariate logistic regression of shunt-dependent hydrocephalus according to the potential predictive factors (adjusted for sex, age group, Hunt-Hess grade, modified Fisher grade, IVH, ICH, EVD, operation type, white blood cell, sodium, potassium, glucose, blood urine nitrogen, creatinine, osmolarity, and albumin). IVH = intraventricular hemorrhage; ICH = intracerebral hemorrhage; EVD = external ventricular drainage.

We identified the potential predictors (p < 0.05) for shunt-dependent hydrocephalus after SAH occurrence using univariate Cox regression analysis (S2 Table). We performed multivariate Cox regression analysis including sex, age, and potential predictive covariates based on the univariate analysis. The independent predictive factors for shunt-dependent hydrocephalus were as follows: (1) age ≧65 years (HR, 0.78; 95% CI, 0.58–1.05; p < 0.001), (2) Hunt-Hess grades 4 and 5 (HR, 2.12; 95% CI, 1.53–2.94; p < 0.001), (3) pan IVH (HR, 1.64; 95% CI, 1.15–2.34; p = 0.007), (4) ICH (HR, 0.55; 95% CI, 0.40–0.76; p < 0.001), (5) sodium > 142.8 (HR, 1.53; 95% CI, 1.13–2.07; p = 0.005), (6) potassium > 3.0 (HR, 0.67; 95% CI, 0.51–0.88; p = 0.003), and (7) glucose > 140.5 (HR, 1.85; 95% CI, 1.34–2.56; p < 0.001) (Fig 5).

## Discussion

The main findings of this study were that high sodium level, low potassium level, and high glucose level from POD 1 to POD 12–16 were related to the V-P shunt in SAH patients with aneurysmal neck clipping. The significant differences in the relevant laboratory parameters (i.e., sodium, potassium, glucose, and osmolarity) between the non-shunt treated group and the shunt-dependent hydrocephalus group emerged at approximately POD 2. The earliest shunt operation was performed at POD 10 and most patients underwent delayed V-P shunt operations in our study. Therefore, we believe that these laboratory parameters may predict shunt-dependent hydrocephalus after clipping for aneurysmal SAH. Advanced age, poor clinical grade at admission, pan IVH, and absence of ICH also increased the risk of receiving a shunt.

### Sodium

Previous studies reported the occurrence of electrolyte imbalance after SAH occurrence.[15–21] Many of these studies reported that hyponatremia after SAH occurrence is more frequent than hypernatremia.[15,17,20] Only some studies noted the occurrence of hypernatremia early after SAH occurrence, which is in agreement with the present study.[16,18] Beseoglu et al.[16] reported the daily variations of serum sodium concentration after SAH occurrence. The authors described that early hypernatremia is associated with poor clinical outcomes. In our study, the patients with SAH presenting modified Fisher grades 2 and 4 and pan IVH at admission tended to show hypernatremia between POD 1 and POD 12–16. This result may be associated with injury to anterior hypothalamic nuclei, predominantly in paraventricular and supraoptic nuclei, caused by perivascular hemorrhage and edema.[16,22] Doshi et al. described that IVH extended into the hypothalamic region in human brain biopsy cases.[22] Damaged hypothalamus leads to hypernatremia due to impaired secretion of arginine-vasopressin (AVP).[23] Reduction of AVP after SAH results in increased serum sodium level.[16,23]

Approximately 90% of total body sodium is present in the extracellular space.[23] Hypernatremia is defined as an elevation in extracellular sodium level and a state of hyperosmolality.[24] In this state, intracellular water shifts out to the extracellular space to maintain equilibrium, resulting in reduction of cellular volume and shrinkage of brain volume. This shrinkage of brain volume may lead to CSF volume expansion due to intracranial hypotension according to the Monro-Kellie hypothesis.[25] Previous studies demonstrated the CSF volume expansions (ventricular enlargement and subdural fluid collection) in hypernatremic state.[26–28] Our study also showed that the patients with high serum sodium levels between POD 1 and POD 12–16 had increased risk to V-P shunt.

### Glucose

Hyperglycemic state after SAH occurrence has been reported as a frequent finding in previous studies.[29–33] In these studies, the patients with hyperglycemia at admission after SAH were reported to be associated with poor clinical outcomes. Hyperglycemia after SAH could be derived from a transient stress reaction (catecholamine releasing) and acute metabolic distress.[29,31,34] Several studies showed that the reason for poor clinical outcome in hyperglycemia patients is associated with systemic vasospasm and delayed cerebral ischemia.[29,31,32,35] The possible association between hyperglycemia and hydrocephalus was described in previous studies.[36–39] Osmolarity consists of sodium, potassium, glucose, and BUN and can be calculated as follows: Osmolarity = 2 Na + 2 K + Glucose/18 + BUN/2.8 (all in mmol/L). Systemic hyperglycemia and hyperosmolar state may lead to fluid shift from the brain parenchyma cells into the extracellular spaces, resulting in ventriculomegaly.[36,37] Osmotic swelling, structural change, and alteration of brain tissue viscoelasticity may occur after prolonged hyperosmolar state. When the brain tissues lose viscoelasticity, the brain could not maintain pressure homeostasis and results in ventriculomegaly.[36–38] In our study, not only hyperglycemia at admission but also prolonged postoperative high glucose levels were significantly related to shunt-dependent hydrocephalus. To the best of our knowledge, this study is one of the few studies that evaluate the association between hyperglycemia and shunt-dependent hydrocephalus in surgically treated SAH patients.

### Potassium

Most body potassium is stored in the intracellular matrix (>98%) and body muscle mass (>60%). The Na+/K+ ATPase maintains a stable proportion of potassium between the intracellular and extracellular spaces.[40,41] Hypokalemia after SAH has been reported in several reports,[40–43] and several possible explanations exist for this result. First, catecholamine surge after SAH, as described above, has an important role in inducing hypokalemic status. When the membrane Na+/K+ ATPase is stimulated by β2-adrenergic activity, potassium shifting occurs from the extracellular space to the intracellular space.^14,33^ Second, the rapid decrease in potassium level from immediate postoperative to POD 1 occurs as a possible result of mannitol administration and urine potassium secretion.[41] The current study found that patients with hypokalemia showed significant increase of shunt-dependent hydrocephalus. This result may be attributed to the absence of direct correlation between hypokalemia and hydrocephalus, but hypokalemia reflects the disease severity.

### Medical managements

In this study, the cut-off value of sodium for predicting shunt-dependent hydrocephalus was 142.8 mEq, which is within the normal range of sodium. Therefore, we think that strict sodium correction may be helpful, not only when the value exceeds the upper limit, but also when it is within the upper normal range (> 142.8 mEq), to prevent shunt-dependent hydrocephalus after SAH. The cut-off value of potassium was 3.0 mEq. We supplied potassium only in response to observed hypokalemia. However, routine use of intravenous saline may also induce renal potassium secretion, which results in hypokalemia after SAH.[44] Therefore, we suggest that correcting potassium imbalance should start in the lower normal range (3.5–4.0 mEq). A previous study also recommended that routine use of potassium with saline for hydration should be considered, rather than only in reaction to hypokalemia.[44] The cut-off value of fasting glucose was 140.5 mg/dL in our study. TPN maintains an adequate nutritional status in acutely ill patients and we routinely used TPN for SAH patients after clipping during their ICU stay. However, previous studies have reported complications of TPN, such as hyperglycemia, infection, and mortality.[45,46] Therefore, we suggested that maintaining the normal serum glucose level using insulin added to the TPN may be helpful for preventing hyperglycemia-induced complications, including potential hydrocephalus occurrence after SAH. Early oral nutrition intake and disuse of TPN should be considered, whenever possible.

### Limitations

This study has several limitations. First, due to the retrospective nature of the present study, findings may be less accurate compared with data from a planned prospective study. Moreover, the single-center study could have limited the generalizability of our findings. Second, the small number of cases may have reduced the statistical power and validation. Third, we only included neurosurgical clipping patients after SAH, which might affect the results. However, a previous meta-study reported that shunt-dependent hydrocephalus did not differ significantly between coiling and clipping.[47] Fourth, due to the retrospective nature, our data were not fully estimated, and we replaced missing data using the multiple imputation method. Missing data are unavoidable in clinical research and multiple imputation is available in standard statistical software, making it possible to handle missing data semi-routinely.[48] A previous study into the accuracy of the multiple imputation method used a small sample of N = 50 and a moderate sample size of N = 100 with missing values ranging from 20% to 50%.[49] Therefore, we think that the sample size (n = 181) included in our study may be tolerable for the multiple imputation in this study. In addition, a previous study reported that estimates derived from the multiple imputation method with an approximately 20% overall missing rate were statistically significant at p < 0.001 and produced the same significance level as the complete data results. The authors also described that any percentage of bias larger than 10% is considered substantial, the multiple imputation method showed the bias in estimates was mostly under 10% in samples with an approximately 20% overall missing rate. Therefore, we think that the findings in the current study may not be significantly different from real data. Finally, time intervals between SAH and shunting in our study appear to higher than in previous studies.[50,51] However, a recent study in Japan indicated that delayed V-P shunting (> 40 days) occurred frequently and that early shunting was significantly associated with coil embolization.[52] Therefore, we think that the relatively higher time intervals between SAH and shunting in our study may be due to the characteristics of our study patients because we only included patients who underwent clipping after aneurysmal SAH.

## Conclusions

Despite the exploratory nature of this study, we found that high sodium, low potassium, and high glucose levels from POD 1 to POD 12–16 were associated with shunt-dependent hydrocephalus after clipping in SAH patients. Our study suggests that daily laboratory measurement should be performed from SAH occurrence to at least one or two weeks. Moreover, strict management of electrolyte imbalance should be considered even if they are within normal ranges. Further large prospective studies are warranted to confirm our findings and elucidate the underlying mechanisms.

## Supporting information

S1 Data All participant data of the study. (XLSX)

S1 Fig Information of missing values in laboratory parameters of the study from preoperation to 28th postoperative day. (TIF)

S2 Fig Information of missing values in laboratory parameters of the study from the first postoperative day to around 14th postoperative day. (TIF)

S3 Fig Variations of various laboratory parameters after subarachnoid hemorrhage occurrence based on shunt dependency: A, white blood cell; B, hemoglobin; C, platelet; D, blood urine nitrogen; E, creatinine; F, albumin.
(TIF)

S4 Fig A. Bar plot showing the association between modified Fisher grade and sodium levels from POD 1 to POD 12–16. B. Bar plot showing the association between IVH and sodium levels from POD 1 to POD 12–16.
(TIF)

S5 Fig Receiver operating characteristic curve for shunt-dependent hydrocephalus after subarachnoid hemorrhage occurrence based on the following factors: A, white blood cell; B, hemoglobin; C, platelet; D, blood urine nitrogen; E, creatinine; F, albumin.
(TIF)

S1 Table Variations of laboratory parameters as time increases in patients with subarachnoid hemorrhage who underwent aneurysmal clipping classified by ventriculo-peritoneal shunt operation. (DOCX)

S2 Table Univariate Cox regression analysis of shunt-dependent hydrocephalus according to various clinical and laboratory factors. (DOCX)
