# Supplementary figures and images for: Early variations of laboratory parameters predicting shunt-dependent hydrocephalus after subarachnoid hemorrhage
Source: PLoS One. 2017 Dec 12;12(12):e0189499. doi: 10.1371/journal.pone.0189499 (PMC5726740; doi:10.1371/journal.pone.0189499)

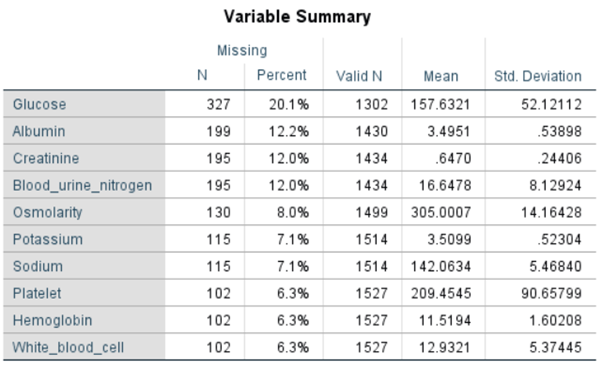

Supplement: S1 Fig — (TIF) [file pone.0189499.s002.tif]

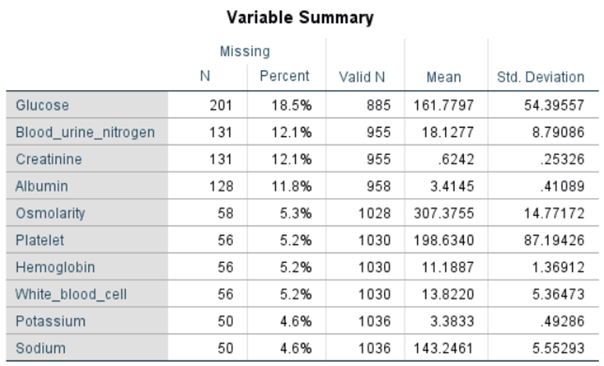

Supplement: S2 Fig — (TIF) [file pone.0189499.s003.tif]

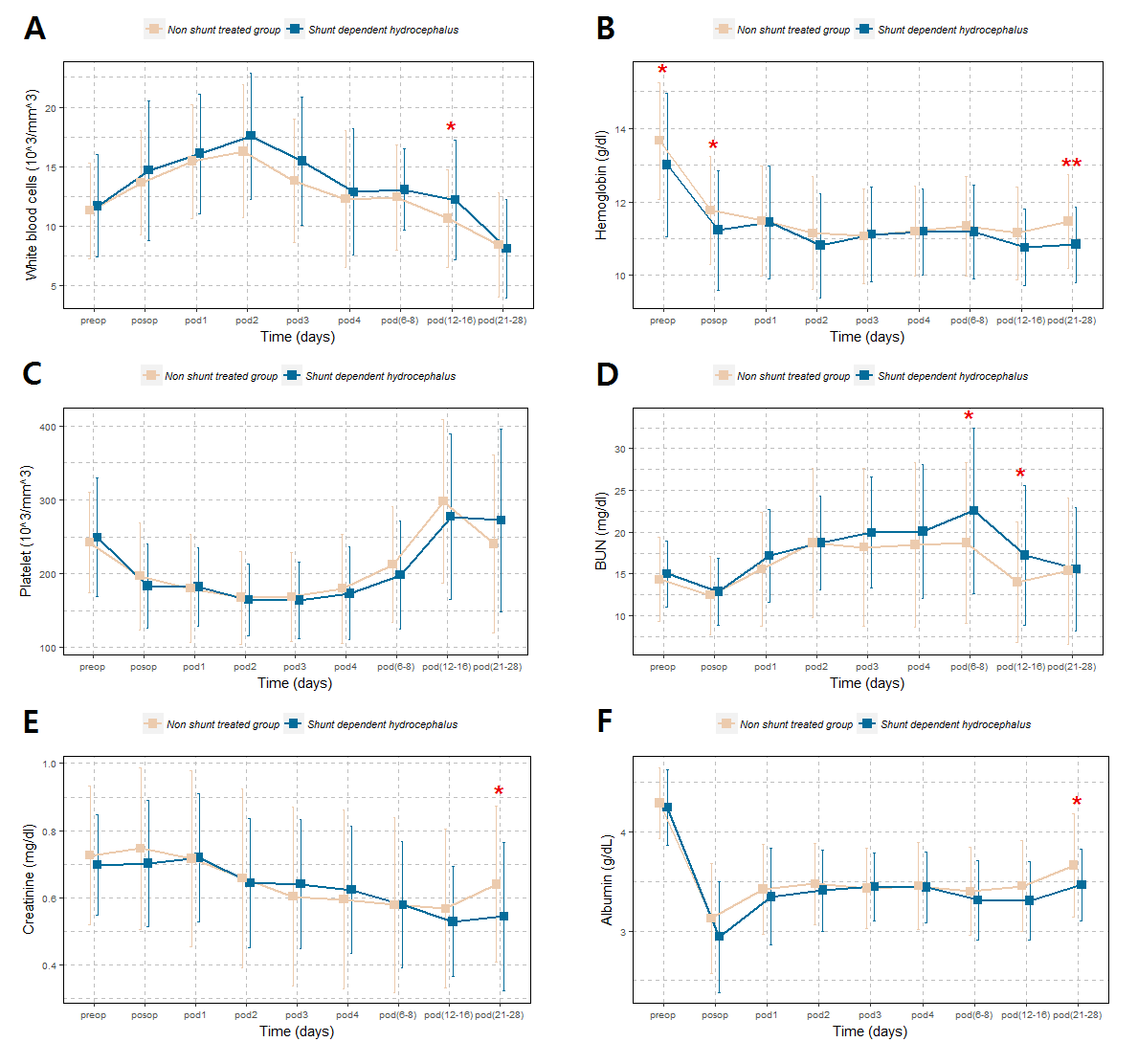

Supplement: S3 Fig — Variations of various laboratory parameters after subarachnoid hemorrhage occurrence based on shunt dependency: A, white blood cell; B, hemoglobin; C, platelet; D, blood urine nitrogen; E, creatinine; F, albumin. (TIF) [file pone.0189499.s004.tif]

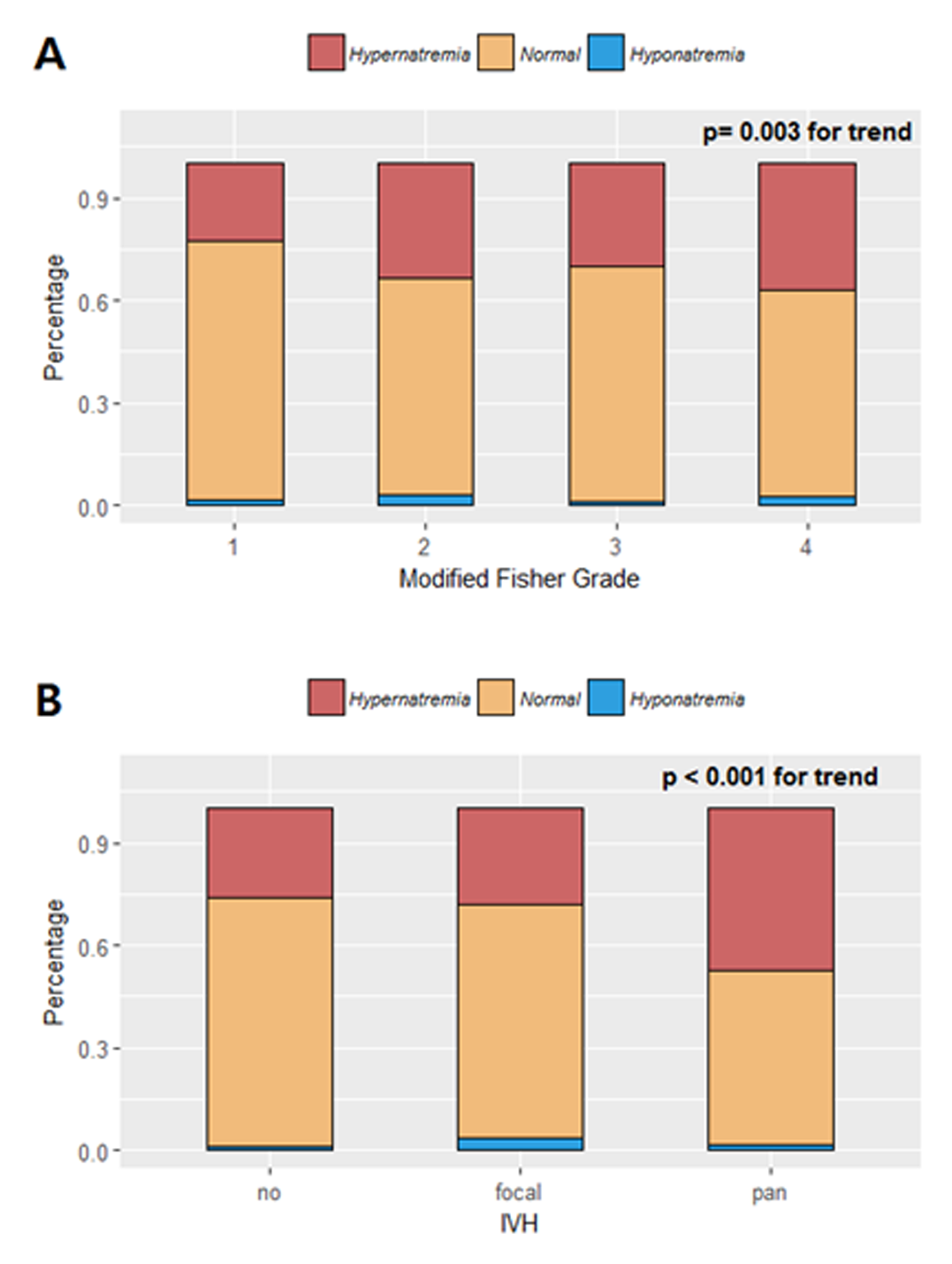

Supplement: S4 Fig — A. Bar plot showing the association between modified Fisher grade and sodium levels from POD 1 to POD 12–16. B. Bar plot showing the association between IVH and sodium levels from POD 1 to POD 12–16. (TIF) [file pone.0189499.s005.tif]

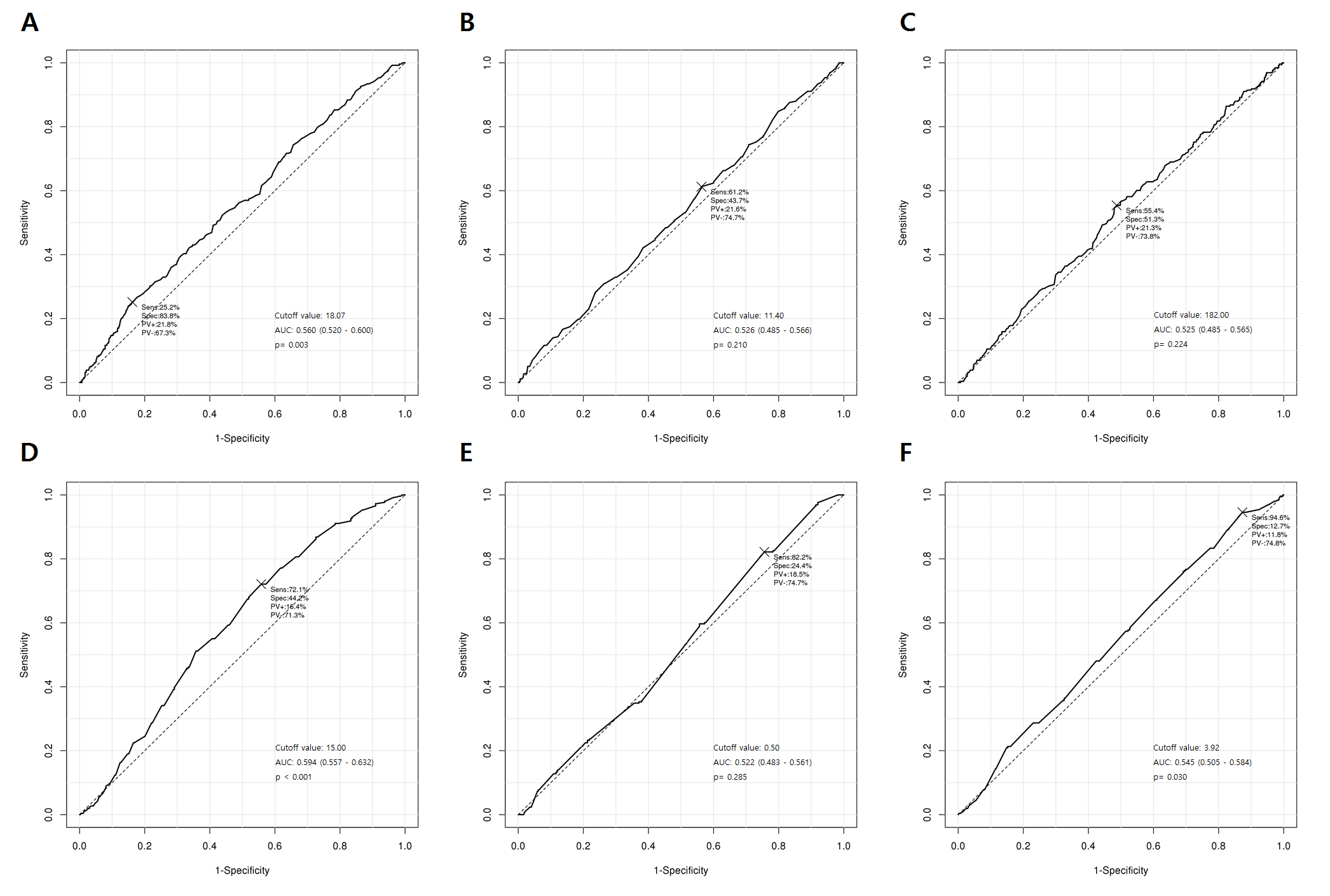

Supplement: S5 Fig — Receiver operating characteristic curve for shunt-dependent hydrocephalus after subarachnoid hemorrhage occurrence based on the following factors: A, white blood cell; B, hemoglobin; C, platelet; D, blood urine nitrogen; E, creatinine; F, albumin. (TIF) [file pone.0189499.s006.tif]
