# Supplementary material for: Early variations of laboratory parameters predicting shunt-dependent hydrocephalus after subarachnoid hemorrhage
Source: PLoS One. 2017 Dec 12;12(12):e0189499. doi: 10.1371/journal.pone.0189499 (PMC5726740; doi:10.1371/journal.pone.0189499)
Supplement: S1 Table — (DOCX) [file pone.0189499.s007.docx]

| Valuables | Shunt | Laboratory data, mean (SD) | | | | | | | | |
| --- | --- | --- | --- | --- | --- | --- | --- | --- | --- | --- |
|  |  | Preparation  (n = 181) | Postoperation  (n = 181) | POD 1  (n = 181) | POD 2  (n = 181) | POD 3  (n = 181) | POD 4  (n = 181) | POD 6–8  (n = 181) | POD 12–16  (n = 181) | POD 21–28  (n = 181) |
| White blood cell (10^3^/mm^3^) | No | 11.3 (4.0) | 13.7 (4.4) | 15.4 (4.8) | 16.3 (5.6) | 13.8 (5.2) | 12.3 (5.8) | 12.4 (4.4) | 10.7 (4.1) | 8.4 (4.4) |
|  | Yes | 11.7 (4.3) | 14.7 (5.9) | 16.1 (5.0) | 17.6 (5.3) | 15.5 (5.4) | 12.9 (5.3) | 13.1 (3.4) | 12.2 (5.0) | 8.1 (4.1) |
| Hemoglobin (g/dl) | No | 13.7 (1.6) | 11.8 (1.5) | 11.5 (1.5) | 11.1 (1.5) | 11.1 (1.3) | 11.2 (1.2) | 11.3 (1.3) | 11.2 (1.3) | 11.5 (1.3) |
|  | Yes | 13.0 (1.9) | 11.2 (1.6) | 11.4 (1.5) | 10.8 (1.4) | 11.1 (1.3) | 11.2 (1.2) | 11.2 (1.3) | 10.8 (1.0) | 10.8 (1.0) |
| Platelet (10^3^/mm^3^) | No | 242.1 (67.7) | 196.8 (72.6) | 180.1 (73.4) | 167.8 (62.8) | 168.6 (60.3) | 179.9 (73.8) | 212.3 (78.1) | 298.0 (111.1) | 240.4 (120.9) |
|  | Yes | 249.1 (80.2) | 183.2 (57.0) | 181.8 (53.2) | 164.5 (48.8) | 163.8 (52.3) | 173.3 (62.9) | 198.3 (73.2) | 277.1 (112.3) | 272.4 (124.0) |
| Sodium (mEq/L) | No | 138.1 (3.2) | 140.4 (3.6) | 141.9 (4.2) | 144.0 (5.1) | 144.1 (5.7) | 143.5 (5.8) | 142.1 (5.4) | 140.0 (4.5) | 140.7 (4.9) |
|  | Yes | 136.8 (6.3) | 139.2 (4.9) | 142.0 (5.5) | 145.9 (5.1) | 146.9 (5.2) | 147.5 (6.9) | 144.9 (5.1) | 142.6 (5.2) | 141.8 (4.5) |
| Potassium (mmol/L) | No | 3.6 (0.4) | 3.9 (0.4) | 3.4 (0.5) | 3.2 (0.4) | 3.3 (0.4) | 3.3 (0.5) | 3.5 (0.5) | 3.9 (0.5) | 3.9 (0.6) |
|  | Yes | 3.4 (0.3) | 3.9 (0.5) | 3.2 (0.5) | 3.0 (0.3) | 3.0 (0.4) | 3.2 (0.4) | 3.5 (0.5) | 3.7 (0.5) | 3.7 (0.6) |
| Glucose (mg/dL) | No | 149.7 (40.7) | 162.6 (40.9) | 188.8 (68.4) | 168.1 (53.0) | 156.0 (4.0) | 150.6 (46.1) | 139.7 (45.2) | 122.7 (42.6) | 128.4 (49.0) |
|  | Yes | 175.0 (63.4) | 174.8 (44.6) | 205.7 (48.2) | 191.0 (49.1) | 186.3 (55.4) | 176.6 (64.8) | 170.9 (47.3) | 154.5 (55.2) | 133.0 (41.2) |
| Blood urine nitrogen (mg/dl) | No | 14.3 (5.0) | 12.5 (4.7) | 15.6 (6.8) | 18.7 (9.0) | 18.2 (9.5) | 18.5 (9.8) | 18.7 (9.6) | 14.0 (7.2) | 15.4 (8.8) |
|  | Yes | 15.0 (3.9) | 12.9 (4.0) | 17.2 (5.6) | 18.7 (5.6) | 19.9 (6.6) | 20.1 (8.0) | 22.6 (9.9) | 17.2 (8.4) | 15.6 (7.4) |
| Creatinine (mg/dl) | No | 0.7 (0.2) | 0.7 (0.2) | 0.7 (0.3) | 0.7 (0.3) | 0.6 (0.3) | 0.6 (0.3) | 0.6 (0.3) | 0.6 (0.2) | 0.6 (0.2) |
|  | Yes | 0.7 (0.2) | 0.7 (0.2) | 0.7 (0.2) | 0.6 (0.2) | 0.6 (0.2) | 0.6 (0.2) | 0.6 (0.2) | 0.5 (0.2) | 0.5 (0.2) |
| Osmolarity (mol/kg) | No | 298.3 (9.7) | 303.1 (10.0) | 308.5 (15.1) | 309.4 (11.8) | 308.5 (13.9) | 307.1 (14.0) | 304.0 (16.6) | 296.4 (11.8) | 298.0 (13.0) |
|  | Yes | 298.4 (14.1) | 301.4 (11.3) | 310.6 (13.4) | 314.7 (12.3) | 313.3 (13.0) | 315.5 (16.3) | 311.8 (14.0) | 304.5 (14.8) | 300.4 (12.8) |
| Albumin  (g/dL) | No | 4.3 (0.4) | 3.1 (0.6) | 3.4 (0.5) | 3.5 (0.4) | 3.4 (0.4) | 3.5 (0.4) | 3.4 (0.4) | 3.5 (0.5) | 3.7 (0.5) |
|  | Yes | 4.2 (0.4) | 2.9 (0.6) | 3.3 (0.5) | 3.4 (0.4) | 3.4 (0.3) | 3.4 (0.4) | 3.3 (0.4) | 3.3 (0.4) | 3.5 (0.4) |

SD, standard deviation; POD, postoperative day
