# Supplementary material for: Early variations of laboratory parameters predicting shunt-dependent hydrocephalus after subarachnoid hemorrhage
Source: PLoS One. 2017 Dec 12;12(12):e0189499. doi: 10.1371/journal.pone.0189499 (PMC5726740; doi:10.1371/journal.pone.0189499)
Supplement: S2 Table — (DOCX) [file pone.0189499.s008.docx]

|  | Univariate Cox regression analysis | | |
| --- | --- | --- | --- |
| Variable | HR | 95% CI | *P* |
| Sex |  |  |  |
| Male | 0.80 | 0.62–1.03 | 0.080 |
| Female |  | Reference |  |
| Age group |  |  |  |
| <65 |  | Reference |  |
| ≥65 | 2.39 | 1.84–3.10 | <0.001 |
| Hunt-Hess grade |  |  |  |
| Grade 1 |  | Reference |  |
| Grade 2 | 1.15 | 0.50–2.65 | 0.740 |
| Grade 3 | 2.85 | 1.25–6.51 | 0.013 |
| Grade 4 | 4.60 | 2.00–10.55 | <0.001 |
| Grade 5 | 4.58 | 1.48–14.22 | 0.008 |
| Modified Fisher grade |  |  |  |
| Grade 1 |  | Reference |  |
| Grade 2 | 1.29 | 0.83–2.02 | 0.261 |
| Grade 3 | 1.17 | 0.76–1.80 | 0.468 |
| Grade 4 | 2.03 | 1.43–2.86 | <0.001 |
| IVH |  |  |  |
| No |  | Reference |  |
| Focal | 1.09 | 0.80–1.50 | 0.575 |
| Pan ventricle | 2.71 | 2.03–3.62 | <0.001 |
| ICH |  |  |  |
| No |  | Reference |  |
| Presence | 0.74 | 0.55–1.00 | 0.047 |
| Aneurysm location |  |  |  |
| Anterior circulation |  | Reference |  |
| Posterior circulation | 0.86 | 0.65–1.15 | 0.310 |
| External ventricular drainage prior to shunt |  |  |  |
| No |  | Reference |  |
| Yes | 3.23 | 2.32–4.51 | <0.001 |
| Operation type |  |  |  |
| Craniotomy |  | Reference |  |
| Craniectomy | 1.84 | 1.38–2.46 | <0.001 |
| Laboratory values |  |  |  |
| WBC, >18.1 (vs ≤ 18.1) | 1.57 | 1.19–2.09 | 0.002 |
| Hemoglobin, >11.4 (vs ≤ 11.4) | 0.93 | 0.72–1.19 | 0.562 |
| Platelet, >182 (vs ≤ 182) | 0.84 | 0.65–1.07 | 0.149 |
| Sodium, >142.8 (vs ≤ 142.8) | 2.40 | 1.85–3.11 | <0.001 |
| Potassium, >3.0 (vs ≤ 3.0) | 0.48 | 0.38–0.62 | <0.001 |
| Glucose, >140.5 (vs ≤ 140.5) | 2.91 | 2.16–3.93 | <0.001 |
| BUN, >15.0 (vs ≤ 15.0) | 1.92 | 1.46–2.52 | <0.001 |
| Creatinine, >0.50 (vs ≤ 0.50) | 1.41 | 1.02–1.93 | 0.036 |
| Osmolarity, >303.0 (vs ≤ 303.0) | 2.57 | 1.92–3.44 | <0.001 |
| Albumin, >3.9 (vs ≤ 3.9) | 0.43 | 0.25–0.75 | 0.002 |

HR, hazard ratio; CI, confidence interval; IVH, intraventricular hemorrhage; ICH, intracerebral hemorrhage; WBC, white blood cell; BUN, blood urine nitrogen
